# Effects of No-Tillage Versus Conventional Tillage and Nitrogen Fertilization on Durum Wheat Grain Quality and Intestinal Cell Responses

**DOI:** 10.3390/plants15213353

**Published:** 2026-10-29

**Authors:** Sonila Alia, Arianna Vignini, Monia Cecati, Mattia Magagnini, Marco Appicciutoli, Marco Bianchini, Biagio di Tella, Marco Fiorentini, Matteo Francioni, Federico Mammarella, Chiara Rivosecchi, Stefano Zenobi, Laura Mazzanti, Elisabetta Giorgini, Roberto Orsini

**Affiliations:** 1 Department of Clinical Sciences, Polytechnic University of Marche, 60126 Ancona, Italy; s.alia@staff.univpm.it (S.A.); l.mazzanti@staff.univpm.it (L.M.); 2 Department for the Promotion of Human Science and Quality of Life, San Raffaele Roma University, 00166 Rome, Italy; monia.cecati@uniroma5.it; 3 Biotecnica Associati Lab, 60022 Castelfidardo, Italy; magagnini@biotecnicaassociati.com; 4 Department of Agricultural, Food and Environmental Sciences, Polytechnic University of Marche, 60131 Ancona, Italy; m.appicciutoli@pm.univpm.it (M.A.); m.bianchini@pm.univpm.it (M.B.); b.ditella@staff.univpm.it (B.d.T.); m.fiorentini@staff.univpm.it (M.F.); m.francioni@staff.univpm.it (M.F.); f.mammarella@pm.univpm.it (F.M.); c.rivosecchi@pm.univpm.it (C.R.); s.zenobi@staff.univpm.it (S.Z.); r.orsini@staff.univpm.it (R.O.); 5 Department of Life and Environmental Sciences, Polytechnic University of Marche, 60131 Ancona, Italy; e.giorgini@staff.univpm.it

**Keywords:** conservation agriculture, oxidative stress, reactive oxygen species, Caco-2 cells, lutein

## Abstract

**Background:** Conservation agriculture has emerged as a sustainable strategy to improve soil quality while maintaining crop productivity and grain nutritional value. However, limited information is available on how agronomic practices influence the biological activity of durum wheat grain. This study, conducted within the framework of the PFRLab project, investigated the effects of no-tillage (NT) and conventional tillage (CT) under two nitrogen fertilization levels (0 and 180 kg ha^−1^ N) on durum wheat grain quality and the biological responses of Caco-2 intestinal epithelial cells. **Methods:** Grain quality was evaluated by determining protein, lutein, and mineral contents. The biological activity of grain extracts was assessed by measuring Caco-2 cell viability using the MTT assay and intracellular oxidative activity using H_2_DCFDA-derived fluorescence after 3, 24, and 48 h of incubation. **Results:** Grain protein and lutein contents tended to be higher under nitrogen fertilization, whereas differences between tillage systems were less consistent for the measured compositional traits. In contrast, distinct responses emerged in the cellular assays. NT-derived extracts generally maintained higher Caco-2 cell viability than CT-derived extracts, particularly after 24 and 48 h, while CT-derived extracts generally elicited higher H_2_DCFDA-derived intracellular fluorescence. Nitrogen fertilization influenced both cellular endpoints, with effects varying according to incubation time and tillage system. Correlation analysis revealed negative but non-significant associations between intracellular fluorescence and cell viability at individual incubation times (r = −0.649 at 3 h, −0.353 at 24 h, and −0.198 at 48 h). **Conclusions:** The findings of this pilot exploratory study suggest that long-term no-tillage can maintain the measured durum wheat grain-quality traits at levels comparable to conventional tillage under Mediterranean conditions, while agronomic management is also associated with differences in the biological activity of grain extracts. The cellular findings provide complementary information rather than evidence of direct health effects or specific chemical mechanisms. Future studies integrating targeted phytochemical characterization of grain extracts, soil microbial analyses, and complementary cellular approaches may help clarify the relationships between agronomic management, grain composition, and biological activity.

## 1. Introduction

With the continued increase in the world population and the mounting pressure of climate change, agricultural systems must intensify production while improving sustainability. Durum wheat (*Triticum turgidum* L. subsp. *durum*) remains a key staple crop, providing essential calories, proteins and micronutrients for millions of people, particularly in the Mediterranean basin, which accounts for a large proportion of global durum cultivation. In this context, Italy is one of the world’s top producers of durum wheat, contributing significantly to global supply [1,2]. 

To meet future food demands sustainably, conservation agriculture (CA) approaches, including no-tillage (NT) and optimized fertilization strategies, have been widely investigated. CA aims to enhance ecological and economic performance by maintaining permanent soil cover, minimizing soil disturbance, and promoting crop diversity. These practices can improve soil health and reduce greenhouse gas emissions by lowering fuel use and enhancing carbon sequestration compared to conventional tillage (CT) systems. Modern research supports these sustainability goals while also examining impacts on crop quality and nutrient dynamics [3,4,5,6].

Several studies demonstrate that tillage systems and fertilization practices strongly influence wheat grain quality traits such as protein concentration, gluten properties, mineral composition, and other nutritional components [7,8]. Under Mediterranean and temperate conditions, CT often increases grain protein concentration relative to NT, likely due to enhanced nitrogen (N) mineralization and nutrient availability in tilled soils. Conversely, NT systems may exhibit slower nitrogen release from surface residues, potentially affecting nitrogen uptake and grain quality parameters [9].

However, these responses are highly context-dependent and influenced by soil fertility status, fertilization regime, and environmental conditions [10].

An additional mechanism through which tillage and nitrogen fertilization may influence crop performance and grain quality involves their long-term effects on soil physical, chemical, and biological properties. Tillage intensity, residue retention, and N availability can modify soil organic matter, nutrient cycling, aeration, and the structure and activity of soil microbial communities, thereby affecting nutrient availability and plant uptake. Evidence from the same long-term experimental site used in the present study showed that NT and CT generated distinct soil physical and chemical conditions and altered active bacterial communities, including microbial groups involved in N transformations. In particular, long-term NT was associated with higher soil organic carbon and total N, whereas differences in soil porosity contributed to the establishment of distinct active bacterial communities [11]. Subsequent research conducted at the same experimental site showed that soil management also influenced durum wheat N and chlorophyll status and confirmed higher soil organic matter and total N under NT than under more intensively tilled conditions. These findings highlight the importance of considering the soil–microbe–plant continuum when evaluating the effects of long-term agronomic management on crop nutritional status and, potentially, on the composition and quality of harvested grain [12].

Specific research on durum wheat has highlighted that NT systems, when properly managed, can improve sustainability without necessarily compromising grain quality, although the response may vary according to climatic conditions and the transition period from conventional to conservation agriculture [13]. Long-term CA practices have been reported to enhance crude protein content, mineral composition, and bioactive compounds of durum wheat grains under Mediterranean conditions, suggesting that soil management affects not only yield but also the nutritional and functional quality of the grain [14,15]. In parallel, fertilization strategies—particularly nitrogen management—remain a key driver of protein accumulation and micronutrient content. Innovative approaches such as optimized nitrogen sources, low-emission fertilization, and fertigation have shown promising effects on nitrogen use efficiency, grain protein content, and bioactive compound accumulation, especially under NT systems [16]. 

Furthermore, innovative fertilization strategies aimed at reducing emissions and optimizing nutrient use (e.g., low-emission nitrogen fertilization) have demonstrated promising results for durum wheat, with improvements in yield, micronutrient content (Fe, Zn), antioxidant activity, and economic sustainability without detriment to grain quality [17,18].

Beyond their nutritional value, durum wheat grains are also a source of bioactive phytochemicals, including phenolic acids, carotenoids, and antioxidant compounds [19], which have been associated with antioxidant and anti-inflammatory biological activities. The concentration and profile of these compounds are known to be influenced by agronomic practices, including tillage and fertilization regimes. However, while compositional analyses provide valuable information, they do not fully capture the biological relevance of such variations.

In this context, in vitro cell-based models provide a useful approach for investigating whether agronomic management is associated with differences in the biological activity of grain-derived compounds. Human Caco-2 intestinal epithelial cells are widely employed as a validated model of the intestinal barrier to assess cell viability, oxidative stress, and bioactivity of cereal-derived compounds. Several studies have demonstrated that wheat grain extracts and phenolic-rich cereal fractions can directly interact with intestinal cells, influencing reactive oxygen species (ROS) production, antioxidant defense mechanisms, and cellular vitality, thereby providing insight into their biological activity [20,21]. Moreover, Caco-2 models have been successfully used to evaluate the bioavailability and biological impact of minerals and antioxidant compounds from wheat-based foods, highlighting their relevance for assessing functional grain quality beyond conventional chemical parameters [22,23].

Despite extensive evidence that long-term tillage and N management can modify soil properties, nutrient dynamics, microbial communities, and crop N status [11,12], it remains unclear whether these agronomic effects are reflected not only in conventional durum wheat grain-quality traits but also in the biological activity of grain-derived compounds. Although Caco-2 models have been used to investigate the biological activity and bioaccessibility of cereal-derived compounds [20,22,23], few studies have integrated long-term agronomic management, grain composition, and intestinal cellular responses within the same experimental framework. Addressing this knowledge gap may provide a more comprehensive understanding of how agronomic management influences durum wheat quality beyond conventional compositional parameters.

Therefore, the present study, conducted within the framework of the PFRLab project “Setting of a precision farming robotic laboratory for cropping system sustainability and food safety and security (PFRLab)” [4], aimed to investigate the effects of long-term NT and CT systems, in interaction with nitrogen fertilization, on durum wheat grain quality under Mediterranean growing conditions. In addition to conventional quality traits, including protein content, lutein concentration, and mineral composition, the biological activity of grain extracts was evaluated in Caco-2 intestinal epithelial cells by assessing cell metabolic viability and H_2_DCFDA-derived intracellular fluorescence. By integrating agronomic, compositional, and cellular analyses within this agronomic framework, the cellular assays were included as complementary endpoints to determine whether differences associated with long-term crop management extended beyond the conventional compositional traits measured in the grain.

## 2. Materials and Methods

### 2.1. Site Description

The field experiment was conducted at the “Pasquale Rosati” experimental farm of Marche Polytechnic University in Agugliano, central Italy (43°32′ N, 13°22′ E; approximately 100 m a.s.l.). The experimental site is located on a hillslope under Mediterranean climatic conditions, on a silty-clay soil classified as a Calcaric Gleyic Cambisol [11,12]. The long-term mean annual precipitation is approximately 838 mm, mainly distributed during autumn and winter. November is the wettest month (93 mm), whereas July is the driest (36 mm). The long-term mean minimum and maximum air temperatures are 11.4 and 20.0 °C, respectively [12]. The site is part of a long-term rainfed field experiment established in 1994 to investigate the effects of contrasting soil tillage and nitrogen fertilization strategies under Mediterranean conditions [11,12,24,25,26].

### 2.2. Experimental Design and Crop Management

The experimental site is part of a long-term field experiment established in 1994 and maintained under rainfed Mediterranean conditions. The cropping system consists of a two-year rotation of durum wheat (*Triticum turgidum* L. subsp. *durum*, cv. Grazia, ISEA) and maize (*Zea mays* L.). The crop rotation was duplicated in two adjacent fields to allow both crops to be present each year. The experiment was arranged according to a split-plot design with two independent field blocks, located in the uphill and downhill areas of the experimental site. Soil tillage was assigned to the main plots, while nitrogen fertilization (0 and 180 kg N ha^−1^) was assigned to the subplots. Main plots were 1500 m^2^, and nitrogen-fertilization subplots were 500 m^2^ [12]. Each combination of tillage system and nitrogen fertilization was represented once in each block, resulting in two independent field replicates per treatment (*n* = 2). For the present study, two contrasting soil-management systems were considered: conventional tillage (CT) and no-tillage (NT). Under CT, the soil was subjected to annual mouldboard ploughing to a depth of approximately 40 cm, followed by double harrowing for seedbed preparation before sowing. Under NT, the soil was left undisturbed, crop residues were retained at the soil surface, weeds were controlled before sowing, and durum wheat was established by direct drilling [11,12]. Durum wheat was sown at a seed rate of 220 kg ha^−1^ with a row spacing of 0.17 m [12]. Weed control in NT was performed before direct drilling using glyphosate at 2.25 kg ha^−1^ of active ingredient. Phosphorus was applied to all treatments at a rate of 70 kg P_2_O_5_ ha^−1^. Two nitrogen fertilization treatments were considered in the present study: N0 (0 kg N ha^−1^) and N180 (180 kg N ha^−1^). N0 represented the unfertilized control, whereas N180 corresponded to the conventional N rate historically adopted in the study area. Nitrogen was supplied as urea (46% N) and split into two equal applications, with 50% of the total N rate applied at each application. The agronomic management described above followed the established protocol of the long-term experimental platform, as previously reported in detail by Pastorelli et al. [11] and Fiorentini et al. [12]. For each agronomic treatment, grain samples were collected from both independent field blocks. These subsamples were collected to represent within-plot variability and were considered nested within their respective field experimental units rather than independent biological replicates.

### 2.3. Determination of Grain Protein Content

Grain extracts were prepared from subsamples collected within each of the two independent field replicates (uphill and downhill) for each agronomic treatment. Subsamples were processed separately, and the resulting measurements were subsequently averaged within each field replicate for statistical analysis. After harvest, grain samples were collected, cleaned to remove foreign material, and stored under dry conditions until analysis. Before protein determination, grain samples were ground using a laboratory mill to obtain a particle size <1 mm (≥90% passing). No additional oven-drying step was performed before analysis. Total nitrogen was determined using the Kjeldahl method according to ICC Standard Method No. 105/2 [27]. Grain protein content was calculated from total nitrogen using a nitrogen-to-protein conversion factor of 5.7 (protein = N × 5.7) and expressed on a dry matter basis.

### 2.4. Determination of Total Lutein

Total lutein was determined by hot saponification followed by solvent extraction, according to the method described by Panfili et al. [28], with minor modifications. Briefly, 2.0 g of finely ground wheat sample were weighed into Pyrex screw-capped tubes. To each tube, 4 mL of butylated hydroxytoluene (BHT, 50 g L^−1^) as antioxidant, 2 mL of ethanol (96%, *v*/*v*), 1 mL of sodium chloride solution (10 g L^−1^), and 2 mL of potassium hydroxide solution (600 g L^−1^) were added. Tubes were tightly capped and placed in a water bath at 70 °C for 45 min with intermittent agitation every 5–10 min to promote saponification. After cooling in an ice bath, 15 mL of sodium chloride solution (100 g L^−1^) was added. The carotenoids were extracted by repeated liquid–liquid extractions using a mixture of n-hexane:ethyl acetate (9:1, *v*/*v*) until the aqueous phase was colorless. The combined organic extracts were evaporated to dryness under reduced pressure using a rotary evaporator, and the residue was re-dissolved in 2 mL of hexane containing 10% (*v*/*v*) isopropyl alcohol. Lutein quantification was performed by high-performance liquid chromatography (HPLC) using an isocratic system. Separation was achieved on a C18 reverse-phase column (Tecknokroma Mediterranea Sea 18, 5 µm, 25 × 0.46 cm). The mobile phase consisted of 0.15% phosphoric acid solution (A) and acetonitrile (B) in a 10:90 (*v*/*v*) ratio, at a flow rate of 1.2 mL min^−1^. The column effluent was monitored at 450 nm using a UV–Vis detector (YL Instruments 9300 HPLC System) over a 15-min runtime. Samples were filtered through a 0.45 µm membrane prior to injection. Lutein peaks were identified and quantified by comparison with an external lutein standard. Calibration curves were constructed in the range of 1–50 µg mL^−1^, with linearity confirmed by R^2^ > 0.999. Each sample was analyzed in triplicate to ensure reproducibility, and lutein content was expressed on a dry weight basis (µg g^−1^ DW).

### 2.5. Determination of Minerals

Mineral concentrations in durum wheat grain were determined using acid digestion followed by ICP-OES analysis. Approximately 0.5 g of ground sample was accurately weighed and transferred into SCP Science digestion tubes. To each tube, 20 mL of aqua regia (a mixture of concentrated nitric acid and ultrapure hydrochloric acid) was added. The samples were digested on a hot plate for 2 h until complete sample decomposition. After cooling, the digest was quantitatively transferred into volumetric flasks and diluted to 50 mL with ultrapure water (18 MΩ·cm). For quality control, a digestion blank was prepared by processing an equal volume of ultrapure water, and a certified reference material was subjected to the same digestion procedure to ensure method accuracy. Mineral concentrations were measured using an Agilent 720 ICP-OES system. Analyses were performed in triplicate, and results were expressed on a dry weight basis (mg kg^−1^ DW).

### 2.6. Caco-2 Cell Culture and Treatment

Human colon adenocarcinoma cells (Caco-2, ATCC HTB-37) were obtained from the American Type Culture Collection (Rockville, MD, USA). Caco-2 cells between passages 10 and 28 were cultured in Dulbecco’s Modified Eagle Medium (DMEM) supplemented with 10% (*v*/*v*) fetal bovine serum (FBS), 1% L-glutamine, 1% penicillin/streptomycin, and 1% non-essential amino acids. Cells were maintained at 37 °C in a humidified atmosphere containing 5% CO_2_. The culture medium was renewed every 2 days, and cells were subcultured by trypsinization upon reaching approximately 80% confluence. For experimental treatments, cells were seeded at a density of 5 × 10^5^ cells per well in 12-well plates and allowed to adhere for 24 h prior to treatment, unless otherwise stated. Based on previous studies of food-derived bioactive compounds in Caco-2 cells, concentrations up to approximately 10 µg/mL have been used without inducing cytotoxicity, whereas higher concentrations (≥25 µg/mL) may elicit dose-dependent cellular effect (e.g., changes in viability or barrier integrity) [29]. Therefore, the present study employed a concentration of 10 µg/mL of durum wheat grain extract to assess its effects on Caco-2 cell metabolic viability and intracellular oxidative activity.

### 2.7. Preparation of Durum Wheat Grain Extracts

Durum wheat grain samples were ground to obtain whole-grain flour. For each sample, 5 g of flour was extracted with 5 mL of deionized water containing 1% (*v*/*v*) ethanol and incubated at 40 °C for 4 h. After extraction, the samples were centrifuged at 5000 rpm for 5 min, and the supernatants were filtered through a 0.45 μm syringe filter to remove particulate material. The filtered extracts were subsequently freeze-dried (lyophilized) and stored at −20 °C until use [30]. Before cell treatment, the lyophilized extracts were reconstituted in phosphate-buffered saline (PBS). Appropriate aliquots were added to the culture medium to obtain a final dry-extract concentration of 10 µg mL^−1^. The extraction procedure was designed to recover water-soluble and mildly polar bioactive compounds while minimizing their degradation during processing.

### 2.8. Cell Viability Assay

Cell viability was evaluated using the MTT (3-(4,5-dimethylthiazol-2-yl)-2,5-diphenyltetrazolium bromide) tetrazolium reduction assay, according to established protocols [31]. Caco-2 cells were seeded at a density of 4 × 10^4^ cells per well in 96-well plates and allowed to adhere overnight at 37 °C in a humidified atmosphere containing 5% CO_2_.

After treatment, the culture medium was removed and cells were incubated with MTT solution (5 mg/mL) for 3 h at 37 °C. The resulting formazan crystals were solubilized by the addition of dimethyl sulfoxide (DMSO), and absorbance was measured at 540 nm using a microplate reader. Cell viability was expressed as a percentage relative to untreated control cells.

### 2.9. Assessment of Intracellular Oxidative Activity

Intracellular oxidative activity was assessed using the fluorescent probe 2′,7′-dichlorodihydrofluorescein diacetate (H_2_DCFDA) [32]. Cells were trypsinized, counted, and seeded at a final density of 4 × 10^4^ cells per well. After treatment, cells were washed with PBS and incubated with H_2_DCFDA (10 µM), freshly diluted from a DMSO stock solution in serum-free medium, for 45 min at 37 °C in the dark. The probe was then removed and cells were washed with PBS prior to fluorescence measurement. The Multi-Mode Microplate Reader SynergyTM HT (BioTek Instruments, Inc., Winooski, VT, USA) was used to measure cell fluorescence from each well. Fluorescence was measured at excitation/emission wavelengths of 485/535 nm. H_2_DCFDA-derived fluorescence was expressed in arbitrary units (a.u.) and interpreted as a non-specific indicator of intracellular oxidative/redox activity rather than as a direct quantitative measurement of reactive oxygen species production.

### 2.10. Statistical Analysis

The field experiment followed a split-plot design with two independent blocks (uphill and downhill), with tillage system (no-tillage, NT; conventional tillage, CT) as the main-plot factor and nitrogen fertilization (0 and 180 kg N ha^−1^) as the subplot factor. For each agronomic treatment, grain subsamples were collected from both field blocks and considered nested within their respective experimental units rather than independent biological replicates. Subsample measurements were averaged within each field replicate, resulting in two independent field-level observations per treatment (n = 2). The availability of the original data differed between grain-composition measurements and cellular assays. For the cellular experiments, individual measurements could be assigned to their respective uphill and downhill field replicates, allowing aggregation at the field experimental-unit level and subsequent split-plot ANOVA. In contrast, the available grain-composition data consisted only of treatment-level means and standard deviations, without the original block-specific observations. Therefore, the split-plot error structure could not be reconstructed for grain protein, lutein, and mineral concentrations, and these variables were evaluated descriptively rather than through inferential statistical comparisons. Data are presented as mean ± standard deviation (SD). For the cellular endpoints, the effects of tillage system, nitrogen fertilization, and their interaction were evaluated using split-plot ANOVA, accounting for the block structure and the different experimental error terms. The main effect of tillage was tested against the block × tillage error term, whereas nitrogen fertilization and the tillage × nitrogen interaction were tested against the subplot error term. Cellular responses were analyzed separately at 3, 24, and 48 h. Results are presented as mean ± SD. The untreated cellular control was excluded from the agronomic factorial analysis and used as a descriptive reference. The relationship between H_2_DCFDA-derived intracellular fluorescence and Caco-2 cell metabolic viability was assessed using Pearson’s correlation analysis based on field-replicate means for each agronomic treatment and incubation time. Correlations were calculated separately for each incubation time, while the overall association across incubation times was considered descriptive because repeated observations from the same field experimental units were not fully independent. Statistical significance was set at *p* < 0.05. Statistical analyses were performed using GraphPad Prism version 11.1.0 (GraphPad Software, San Diego, CA, USA).

## 3. Results

### 3.1. Effects of Tillage System and Nitrogen Fertilization on Grain Quality

Grain protein content, lutein concentration, and mineral composition showed different patterns across tillage systems and nitrogen fertilization levels (Table 1). Under unfertilized conditions (0 kg ha^−1^ N), protein content was slightly higher in no-tillage (NT) wheat (13.17 ± 1.34%) compared to conventional tillage (CT) (12.65 ± 1.23. At 180 kg ha^−1^ N, protein content increased in both tillage systems, reaching 14.24 ± 1.41% in NT and 14.20 ± 1.43% in CT.

Lutein concentration followed a similar pattern. At 0 kg ha^−1^ N, lutein content was 0.17 ± 0.01 µg g^−1^ DW in NT and 0.15 ± 0.02 µg g^−1^ DW in CT. Lutein concentration was higher under nitrogen fertilization in both tillage systems, reaching 0.21 ± 0.03 µg g^−1^ DW in NT and 0.18 ± 0.02 µg g^−1^ DW in CT at 180 kg ha^−1^ N.

Regarding mineral composition, copper (Cu) content at 0 kg ha^−1^ N was 2.65 ± 0.23 mg kg^−1^ in NT and 3.37 ± 0.31 mg kg^−1^ in CT, while at 180 kg ha^−1^ N values increased to 3.35 ± 0.32 mg kg^−1^ in NT and 3.51 ± 0.35 mg kg^−1^ in CT.

Manganese (Mn) concentration was consistently higher under CT than NT at both nitrogen levels. At 0 kg ha^−1^ N, Mn content was 9.22 ± 0.91 mg kg^−1^ in NT and 14.25 ± 1.26 mg kg^−1^ in CT, while at 180 kg ha^−1^ N it was 8.64 ± 0.83 mg kg^−1^ in NT and 12.80 ± 1.06 mg kg^−1^ in CT.

Selenium (Se) content showed low values under unfertilized conditions (0.03 ± 0.004 mg kg^−1^ in NT and 0.06 ± 0.007 mg kg^−1^ in CT) and slightly higher values at 180 kg ha^−1^ N (0.04 ± 0.005 mg kg^−1^ in NT and 0.04 ± 0.006 mg kg^−1^ in CT).

Overall, nitrogen fertilization tended to increase protein content and lutein concentration, whereas mineral composition showed element-specific trends.

### 3.2. Effects of Tillage System and Nitrogen Fertilization on Caco-2 Cell Viability

**Figure 1:**
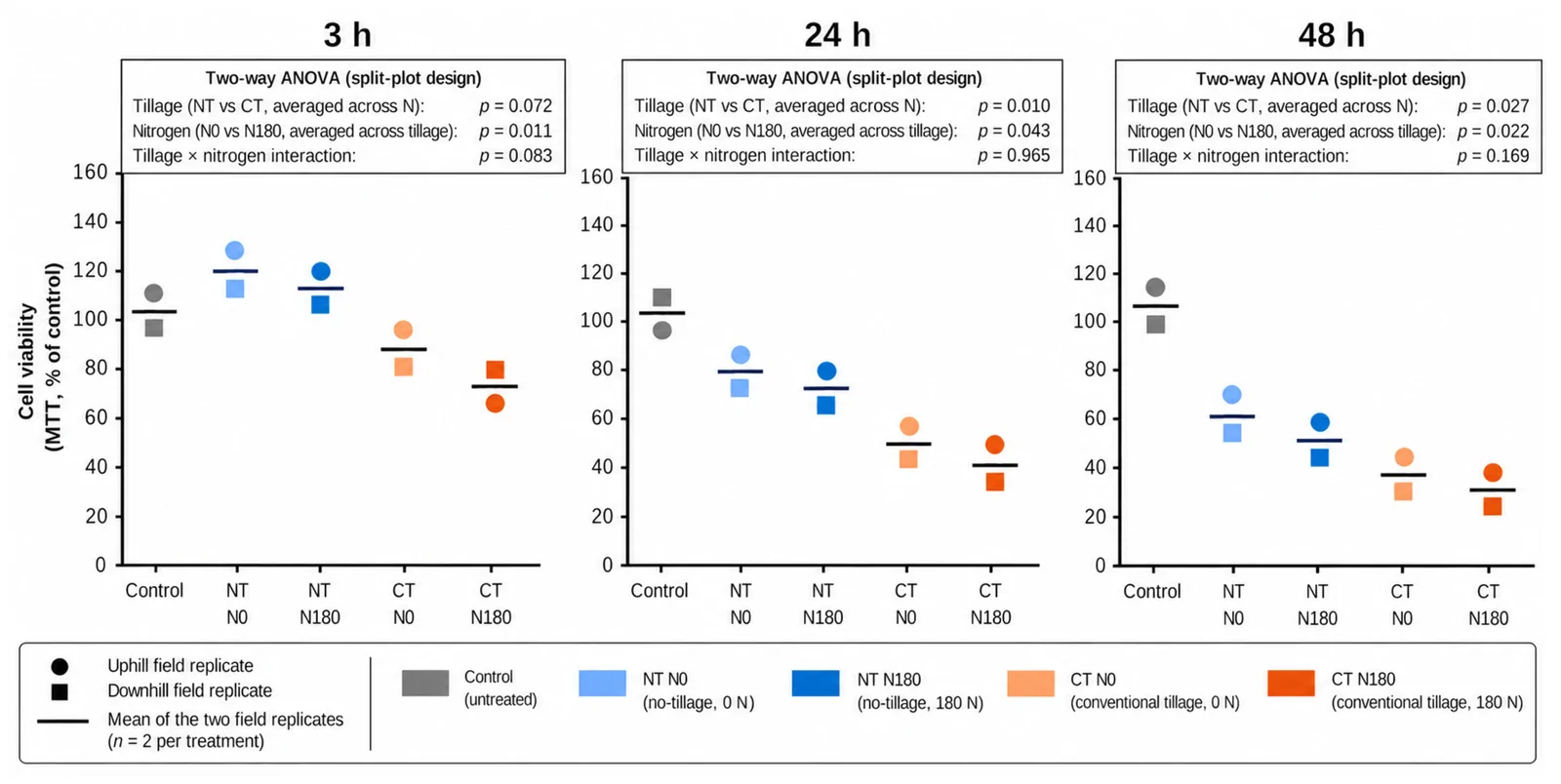
Effects of tillage system and nitrogen fertilization on Caco-2 cell metabolic viability following exposure to durum wheat grain extracts for 3, 24, and 48 h. Treatments included no-tillage (NT) and conventional tillage (CT), each under two nitrogen fertilization levels (N0, 0 kg N ha^−1^; N180, 180 kg N ha^−1^). Circles and squares represent the uphill and downhill field replicates, respectively, while horizontal bars indicate the mean of the two independent field replicates (n = 2 per treatment). Untreated cells are shown as a separate control group and were excluded from the analysis. The reported *p*-values indicate the main effects of tillage (NT vs. CT, averaged across nitrogen levels), nitrogen fertilization (N0 vs. N180, averaged across tillage systems), and their interaction (tillage × nitrogen) at each incubation time.

Caco-2 cell metabolic viability varied according to incubation time and the agronomic management of durum wheat (Figure 1). Overall, extracts obtained under no-tillage (NT) tended to maintain higher cell viability than those obtained under conventional tillage (CT), although statistical significance of the treatment effects varied over time.

After 3 h of incubation, NT-derived extracts were associated with higher cell viability than CT-derived extracts. However, the main effect of tillage did not reach statistical significance (*p* = 0.072). Nitrogen fertilization significantly affected cell viability (*p* = 0.011), whereas the tillage × nitrogen interaction was not significant (*p* = 0.083).

After 24 h, cell viability was lower in all extract-treated groups than in the untreated control. Significant main effects were observed for both tillage (*p* = 0.010) and nitrogen fertilization (*p* = 0.043), whereas their interaction was not significant (*p* = 0.965). NT-derived extracts generally maintained higher viability than CT-derived extracts.

After 48 h, cell viability decreased further in all extract-treated groups. NT-derived extracts continued to show higher viability than CT-derived extracts. Significant effects were detected for tillage (*p* = 0.027) and nitrogen fertilization (*p* = 0.022), while their interaction remained non-significant (*p* = 0.169).

Overall, these findings indicate that both tillage system and nitrogen fertilization were associated with differences in Caco-2 metabolic viability, with the effects becoming more evident after prolonged exposure. NT-derived extracts generally maintained higher cell viability than CT-derived extracts, particularly at 24 and 48 h.

### 3.3. Effects of Tillage System and Nitrogen Fertilization on Intracellular Oxidative Activity in Caco-2 Cells

**Figure 2:**
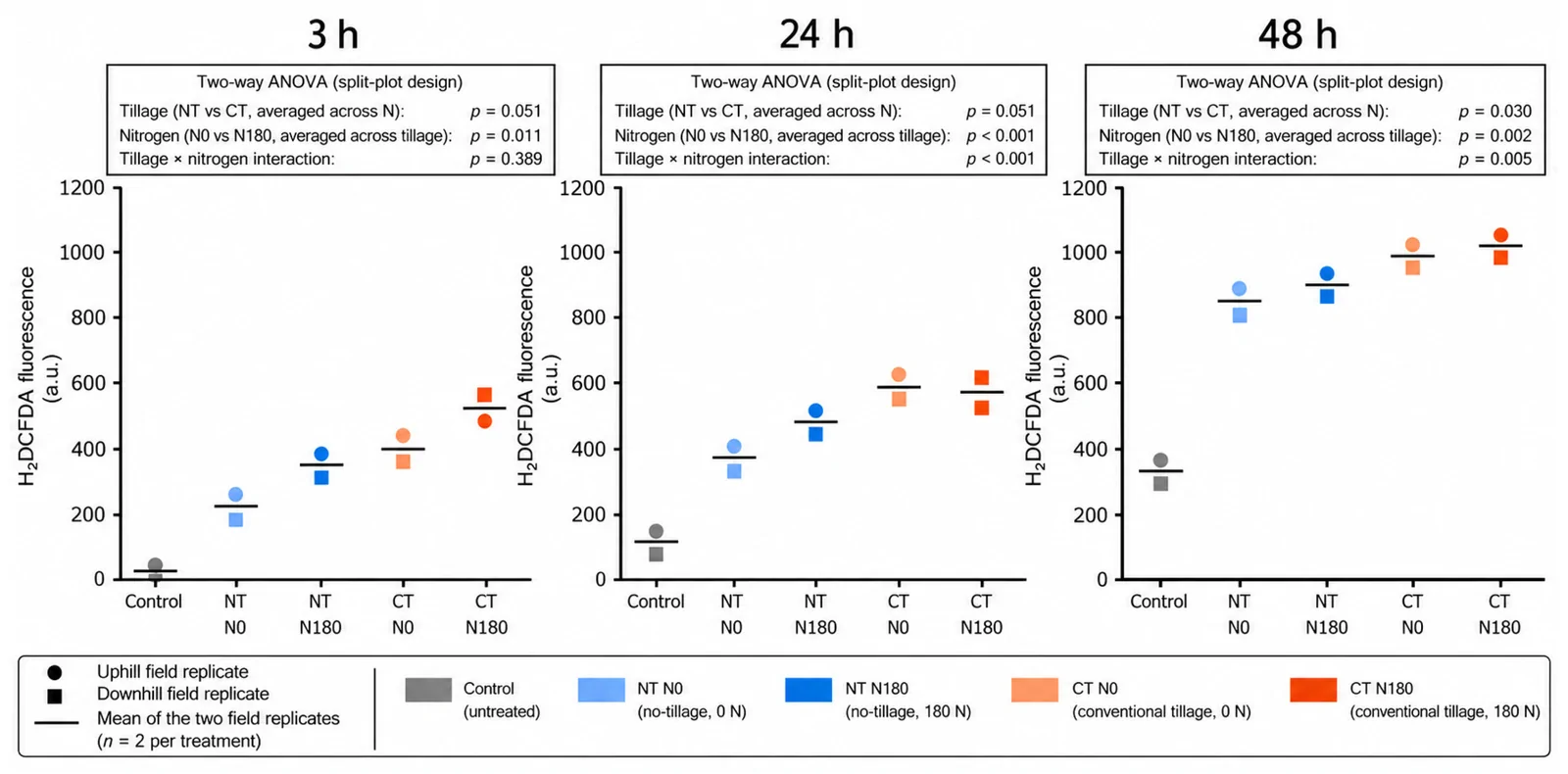
Effects of tillage system and nitrogen fertilization on H_2_DCFDA-derived intracellular fluorescence (a.u.) in Caco-2 cells exposed to durum wheat grain extracts for 3, 24, and 48 h. Treatments included no-tillage (NT) and conventional tillage (CT), each under two nitrogen fertilization levels (N0, 0 kg N ha^−1^; N180, 180 kg N ha^−1^). Circles and squares represent the uphill and downhill field replicates, respectively, and horizontal bars indicate the mean of the two independent field replicates (n = 2 per treatment). Untreated controls are shown separately and were excluded from the factorial analysis. The reported *p*-values indicate the main effects of tillage (NT vs. CT, averaged across nitrogen levels), nitrogen fertilization (N0 vs. N180, averaged across tillage systems), and their interaction (tillage × nitrogen) at each incubation time.

Exposure of Caco-2 cells to durum wheat grain extracts resulted in increased H_2_DCFDA-derived intracellular fluorescence, with responses varying according to incubation time, tillage system, and nitrogen fertilization (Figure 2).

After 3 h of incubation, all grain-extract treatments exhibited higher fluorescence values than the untreated control. Among the agronomic treatments, NT–N0 showed the lowest fluorescence, whereas CT–N180 showed the highest. Although CT-derived extracts generally exhibited higher fluorescence than NT-derived extracts, the main effect of tillage did not reach statistical significance (*p* = 0.051). Nitrogen fertilization significantly affected intracellular fluorescence (*p* = 0.001), while the tillage × nitrogen interaction was not significant (*p* = 0.389).

After 24 h, H_2_DCFDA-derived fluorescence increased in all experimental groups. The lowest fluorescence among the agronomic treatments was observed in NT–N0, followed by NT–N180, CT–N180, and CT–N0. The main effect of tillage remained just above the threshold for statistical significance (*p* = 0.051), whereas nitrogen fertilization (*p* < 0.001) and the tillage × nitrogen interaction (*p* < 0.001) were significant. The interaction reflected different responses to nitrogen fertilization under the two tillage systems, with fluorescence increasing under NT but showing a slight reduction under CT.

After 48 h, intracellular fluorescence reached its highest values across all treatments. NT–N0 continued to exhibit the lowest fluorescence among the agronomic treatments, followed by NT–N180, CT–N0, and CT–N180. Significant effects were detected for tillage (*p* = 0.030), nitrogen fertilization (*p* = 0.002), and their interaction (*p* = 0.005). The significant interaction indicated that the response to nitrogen fertilization differed between the two tillage systems.

Overall, H_2_DCFDA-derived fluorescence increased with incubation time, and CT-derived extracts generally elicited higher fluorescence than NT-derived extracts. Nitrogen fertilization influenced the cellular oxidative response, although its effect depended on tillage system and incubation time. These findings indicate that agronomic management was associated with differences in intracellular oxidative/redox activity, as assessed by H_2_DCFDA fluorescence.

### 3.4. Relationship Between H_2_DCFDA-Derived Intracellular Fluorescence and Caco-2 Cell Viability

The relationship between H_2_DCFDA-derived intracellular fluorescence and Caco-2 cell metabolic viability was investigated using Pearson’s correlation analysis (Table 2).

At 3 h, a moderate negative correlation was observed between intracellular fluorescence and cell viability (r = −0.649, R^2^ = 0.421), although the association did not reach statistical significance (*p* = 0.082). At 24 h, the negative association was weaker (r = −0.353, R^2^ = 0.125; *p* = 0.391), and a further reduction in correlation strength was observed at 48 h (r = −0.198, R^2^ = 0.039; *p* = 0.637). When observations from all incubation times were pooled, a strong inverse correlation was observed (r = −0.821, R^2^ = 0.674; nominal *p* < 0.001).

Overall, the results indicate a negative association between H_2_DCFDA-derived fluorescence and Caco-2 metabolic viability, although the correlations observed at individual incubation times were not statistically significant.

## 4. Discussion

The present study investigated the effects of NT and CT systems under two nitrogen fertilization levels (N0, 0 kg N ha^−1^; N180, 180 kg N ha^−1^) on durum wheat grain quality and the biological responses of Caco-2 intestinal epithelial cells to grain extracts. In addition to conventional compositional traits, the study explored the cellular responses of Caco-2 cells to whole-grain extracts, providing a functional perspective on how agronomic management may influence the redox-related biological activity of durum wheat grain extracts under Mediterranean conditions.

Consistent with previous studies, grain protein content and lutein concentration tended to be higher under nitrogen fertilization, whereas differences between tillage systems were less consistent at comparable nitrogen levels. Nitrogen availability is known to strongly regulate nitrogen assimilation and storage protein synthesis in durum wheat grain, particularly under Mediterranean soils where nitrogen mineralization is often a limiting factor [3,16]. The relatively similar protein and lutein values observed under NT and CT at comparable nitrogen levels are consistent with evidence from long-term conservation agriculture experiments indicating that, after an initial transition phase, NT systems can maintain grain quality at levels comparable to CT [10,14]. These findings are consistent with long-term Mediterranean experiments showing that the effects of conservation tillage on grain yield and quality are strongly influenced by environmental conditions and crop management [33].

The relatively high N rate used in the N180 treatment (180 kg N ha^−1^) may also have contributed to reducing differences between tillage systems. Under NT, reduced soil disturbance and surface retention of crop residues can slow organic matter decomposition and N mineralization compared with CT, potentially limiting short-term N availability. Therefore, the external N supply provided by the N180 treatment may have partially compensated for this constraint, contributing to the comparable grain-quality traits observed between NT and CT at the same fertilization level [12]. However, this mechanism was not directly assessed in the present study and should therefore be considered a plausible interpretation rather than a demonstrated causal relationship.

Similarly, lutein concentration tended to be higher under nitrogen fertilization, whereas differences between tillage systems were relatively small. Carotenoid biosynthesis in durum wheat has been linked to nitrogen status and photosynthetic capacity during grain filling, while soil disturbance per se appears to play a secondary role once soil fertility has stabilized [15,17]. The lutein concentrations measured in the present study were lower than those commonly reported for durum wheat grain. Such differences may reflect cultivar-specific characteristics, environmental conditions, or methodological factors affecting carotenoid quantification. However, the present data do not allow the relative contribution of these factors to be determined, and comparisons with previous studies should therefore be interpreted cautiously. Overall, these descriptive findings suggest that conservation tillage may maintain the measured nutritional traits of durum wheat grain under the conditions investigated.

Mineral composition exhibited element-specific patterns, with manganese showing consistently higher mean values under CT than under NT at both nitrogen fertilization levels. Mineral uptake and grain loading are controlled by complex soil–plant interactions, including nutrient availability, root distribution, and translocation efficiency [18]. In long-term tillage experiments, such processes may become buffered over time, reducing detectable differences between soil management systems [14]. Overall, the compositional data suggest that nitrogen fertilization may contribute to variations in grain-quality traits, while NT appears capable of maintaining several measured nutritional characteristics at levels comparable to CT under Mediterranean conditions. This agrees with previous Mediterranean studies reporting that no-tillage can maintain grain quality comparable to conventional tillage once the production system has reached a stable equilibrium [13].

In addition to direct effects on nutrient availability, long-term tillage and N management may indirectly influence grain quality through changes in the soil–microbe–plant continuum. Previous research conducted at the same long-term experimental site showed that NT and CT differently affected soil physical and chemical properties and active soil bacterial communities, including microbial groups involved in N transformations [11]. Moreover, higher soil organic matter and total N under NT have been previously associated with differences in durum wheat N status at this experimental site [12]. Although soil microbial communities were not investigated in the present study, these findings suggest a plausible pathway through which long-term soil management may influence nutrient cycling, plant N acquisition and, ultimately, grain composition. Future studies integrating soil microbial profiling with soil nutrient dynamics and targeted grain metabolomics could help clarify these relationships.

Exposure to durum wheat grain extracts was associated with a time-dependent reduction in Caco-2 cell metabolic viability. NT-derived extracts generally maintained higher viability than CT-derived extracts, with significant tillage effects observed at 24 and 48 h. Nitrogen fertilization significantly influenced cell viability at all three incubation times. H_2_DCFDA-derived intracellular fluorescence increased with incubation time and was generally higher in cells exposed to CT-derived extracts. The main effect of tillage was significant at 48 h, whereas nitrogen fertilization significantly affected fluorescence at all incubation times. Significant tillage × nitrogen interactions were also observed at 24 and 48 h, indicating that the response to nitrogen fertilization depended on the tillage system. These findings indicate that agronomic management was associated with differences in the redox response induced by the grain extracts. 

Although grain extracts obtained under different tillage systems elicited distinct cellular responses, the chemical constituents responsible for these differences remain unidentified. In particular, lutein was quantified following saponification and organic solvent extraction, whereas the extracts used for cellular treatments were prepared using deionized water containing 1% ethanol. Given the limited water solubility of lutein, its contribution to the observed cellular responses cannot be established from the present data. These responses may instead reflect differences in water-soluble or mildly polar constituents, including soluble phenolic compounds and other redox-active molecules. However, these compounds were not directly characterized in the cellular extracts [20,34,35,36]. Further characterization of the actual extracts, including soluble phenolic content, antioxidant capacity, and targeted phytochemical profiling, would help clarify the chemical basis of the observed biological activity.

Correlation analysis revealed negative associations between H_2_DCFDA-derived intracellular fluorescence and MTT-derived cell viability at all incubation times, although none of these associations reached statistical significance when evaluated separately. The pooled dataset showed a strong inverse association, which should be interpreted cautiously because it may partly reflect differences between incubation times. These findings support the complementary interpretation of fluorescence and metabolic viability as distinct cellular endpoints, without establishing a direct causal relationship [22,37]. The observed differences suggest that cellular assays may provide additional information when evaluating the biological properties of grain extracts obtained under contrasting agronomic management systems. The results of the present study should be considered in light of some limitations. First, only one extract concentration was tested, selected on the basis of concentrations previously reported in the literature for comparable cereal-derived treatments in Caco-2 models. While this approach ensured biological relevance, it may have limited the assessment of concentration-dependent cellular responses. Second, the absence of simulated gastrointestinal digestion may have influenced the bioaccessibility of antioxidant compounds and minerals, potentially underestimating treatment-specific effects. Third, only metabolic viability and H_2_DCFDA-derived intracellular fluorescence were assessed, without a specific positive oxidative-stress control. Additional endpoints, including antioxidant enzyme activity, inflammatory markers, and epithelial barrier integrity, would provide further mechanistic information. An additional limitation is that the original block-specific grain-composition data were unavailable, preventing split-plot inferential analysis of these traits, whereas field-replicate information was retained for the cellular assays. Future research should integrate detailed kernel phenotyping and targeted profiling of phenolic acids, carotenoids, and other redox-active compounds with in vitro digestion models, dose–response experiments, and complementary cellular endpoints. Such an integrated approach could help clarify whether physical and compositional differences in the grain contribute to the distinct cellular responses observed between NT and CT.

## 5. Conclusions

In conclusion, long-term no-tillage maintained the measured durum wheat grain-quality traits at levels comparable to conventional tillage under Mediterranean conditions, while grain protein and lutein contents tended to be higher under nitrogen fertilization. These findings support the potential of conservation tillage to preserve durum wheat grain quality under the conditions investigated.

The complementary cellular analyses showed that grain extracts obtained under different tillage systems elicited distinct in vitro responses, with NT-derived extracts generally associated with higher Caco-2 cell metabolic viability and lower H_2_DCFDA-derived intracellular fluorescence than CT-derived extracts. These cellular responses should be regarded as additional descriptors of grain-extract biological activity rather than as evidence of direct health effects, particularly because the specific grain components responsible for these differences were not identified.

Overall, the present pilot exploratory study provides preliminary evidence supporting the hypothesis that long-term tillage and nitrogen management may influence durum wheat grain properties beyond the compositional traits measured, potentially contributing to the different in vitro responses observed in Caco-2 cells. Future research combining soil and rhizosphere microbial characterization with targeted phytochemical profiling of grain extracts, simulated gastrointestinal digestion, dose–response experiments, and additional cellular endpoints could help clarify the relationships between long-term agronomic management, grain composition, and biological properties. Such integrated approaches may contribute to the development of sustainable cropping strategies aimed at preserving both agronomic performance and grain quality.

## Figures and Tables

**Table 1:** Protein content, lutein concentration, and mineral composition of durum wheat grain under no-tillage (NT) and conventional tillage (CT) at two nitrogen fertilization levels (N0, 0 kg N ha^−1^; N180, 180 kg N ha^−1^). Data are presented as mean ± standard deviation (SD). The values describe the observed differences among agronomic treatments; inferential comparisons based on the original split-plot experimental units could not be reconstructed from the available aggregated data.

Treatment	Protein Content (%)	Lutein (µg g^−1^ DW)	Mineral (mg/kg)		
			Cu	Se	Mn
**NT** (0 Kg ha^−1^ N)	13.17 ± 1.34	0.17 ± 0.01	2.65 ± 0.23	0.03 ± 0.004	9.22 ± 0.91
CT (0 Kg ha^−1^ N)	12.65 ± 1.23	0.15 ± 0.02	3.37 ± 0.31	0.06 ± 0.007	14.25 ± 1.26
**NT** (180 Kg ha^−1^ N)	14.24 ± 1.41	0.21 ± 0.03	3.35 ± 0.32	0.04 ± 0.005	8.64 ± 0.83
CT (180 Kg ha^−1^ N)	14.20 ± 1.43	0.18 ± 0.02	3.51 ± 0.35	0.04 ± 0.006	12.8 ± 1.06

**Table 2:** Pearson’s correlation coefficients between H_2_DCFDA-derived intracellular fluorescence and Caco-2 cell metabolic viability at different incubation times. Correlations were calculated using the mean values of the two independent field replicates (uphill and downhill) for each agronomic treatment. R^2^ represents the coefficient of determination. The overall correlation includes observations from all three incubation times.

Time	Pearson’s *r*	*R* ^2^	*p*
3 h	−0.649	0.421	0.082
24 h	−0.353	0.125	0.391
48 h	−0.198	0.039	0.637
Overall	−0.821	0.674	<0.001

## Data Availability

The data presented in this study are available from the corresponding author upon reasonable request.

## Abbreviations

The following abbreviations are used in this manuscript

CA	Conservation agriculture
NT	No-tillage
CT	Conventional tillage
PFRLab	Precision Farming Robotic Laboratory
ROS	Reactive oxygen species
MTT	3-(4,5-Dimethylthiazol-2-yl)-2,5-diphenyltetrazolium bromide
H_2_DCFDA	2′,7′-Dichlorodihydrofluorescein diacetate
DMEM	Dulbecco’s Modified Eagle Medium
FBS	Fetal bovine serum
PBS	Phosphate-buffered saline
DMSO	Dimethyl sulfoxide
BHT	Butylated hydroxytoluene
HPLC	High-performance liquid chromatography
ICP-OES	Inductively coupled plasma optical emission spectrometry
DW	Dry weight
ICC	International Association for Cereal Science and Technology
